# COVID-19, masks and communication in the operating theatre: the importance of face value

**DOI:** 10.1017/S0033291720003669

**Published:** 2020-09-17

**Authors:** Shazia P. Sharif, Elisabeth Blagrove

**Affiliations:** 1 The Royal London Hospital, London, UK; 2 University of Warwick, Coventry, UK

Dear Editors,

We write in response to the study by Zhang et al. (2020) ‘COVID-19 pandemic: study on simple, easy, and practical relaxation techniques while wearing medical protective equipment’. The study team has actively investigated potential anxiety-relieving techniques that healthcare professionals can utilise while working and this is commendable. We want to highlight another area that will impact communication, levels of anxiety and fear – medical protective equipment.

Surgical masks have been worn in operating theatres for over a century as outlined by Zhou, Sivathondan, and Handa (2015). The early impetus to wear masks was to prevent the patient's wound becoming contaminated, leading to subsequent infection. More recently, there has been recognition that masks confer some degree of protection to the surgeon in the event of fluid splash. And now with the potential presence of SARS CoV-2, masks in theatre and other clinical settings are an absolute necessity. An et al. (2020) report that surgeons' have increased feelings of fear at work associated with surgical mask shortage during the pandemic.

The more constrictive FFP3 masks provide protection for the wearer, as well as the patient, *and* other staff. However, the FFP3 mask is uncomfortable, very tight fitting and muffles the voice. In addition, the surgeon concurrently wears a visor. Combination of these extra protective barriers can be cumbersome and tiring over long period of operations. The mask, in particular, can cause difficulties in communication. This is especially important when other non-verbal communication channels (e.g. gesture, vocalisation features and posture) are also constrained by the surgical context.

Most obviously, masks obscure the mouth and reduce effective signalling of essential non-verbal cues. Green and Phillips (2004) demonstrated that both eye and mouth facial regions are typically scanned to ascertain facial expression, however obscuring mouths can also be problematic. First, although muscle activation around the mouth provides more obvious emotional messaging (e.g. smiling, grimacing, etc.), this also provides less reliable and/or authentic signals. Second, although the eye region conveys more nuanced affect, it can also be harder to ‘read’ with certainty, as highlighted by Fox and Damjanovic (2006) and Baron-Cohen, Wheelwright, Hill, Raste, and Plumb (2001). This may be impacted further by a protective visor.

In the operating theatre, and other clinical contexts, surgeons need to be mindful of the impact that wearing masks and visors have on communication. An increased awareness of the potential for miscommunication will enable mitigation against it. Healthcare professionals must be prepared to check instructions have been understood by repeating back, especially during a stressful procedure. We advocate multi-disciplinary research and collaboration between psychologists and medical professionals to explore barriers and solutions in the clinical setting.
